# PKGIα is activated by metal-dependent oxidation *in vitro* but not in intact cells

**DOI:** 10.1016/j.jbc.2022.102175

**Published:** 2022-06-22

**Authors:** Sahar Aminzai, Tingfei Hu, Renate B. Pilz, Darren E. Casteel

**Affiliations:** Department of Medicine, University of California, San Diego, La Jolla, California, USA

**Keywords:** protein kinase G, redox regulation, hydrogen peroxide, cell signaling, cardiovascular, H_2_O_2_, hydrogen peroxide, HRP, horseradish peroxidase, PKGI, Type I cGMP-dependent protein kinase, sGC, soluble guanylate cyclase, VASP, vasodilator-stimulated phosphoprotein

## Abstract

Type I cGMP-dependent protein kinases (PKGIs) are important components of various signaling pathways and are canonically activated by nitric oxide– and natriuretic peptide–induced cGMP generation. However, some reports have shown that PKGIα can also be activated *in vitro* by oxidizing agents. Using *in vitro* kinase assays, here, we found that purified PKGIα stored in PBS with Flag peptide became oxidized and activated even in the absence of oxidizing agent; furthermore, once established, this activation could not be reversed by reduction with DTT. We demonstrate that activation was enhanced by addition of Cu^2+^ before storage, indicating it was driven by oxidation and mediated by trace metals present during storage. Previous reports suggested that PKGIα Cys^43^, Cys^118^, and Cys^196^ play key roles in oxidation-induced kinase activation; we show that activation was reduced by C118A or C196V mutations, although C43S PKGIα activation was not reduced. In contrast, under the same conditions, purified PKGIβ activity only slightly increased with storage. Using PKGIα/PKGIβ chimeras, we found that residues throughout the PKGIα-specific autoinhibitory loop were responsible for this activation. To explore whether oxidants activate PKGIα in H9c2 and C2C12 cells, we monitored vasodilator-stimulated phosphoprotein phosphorylation downstream of PKGIα. While we observed PKGIα Cys^43^ crosslinking in response to H_2_O_2_ (indicating an oxidizing environment in the cells), we were unable to detect increased vasodilator-stimulated phosphoprotein phosphorylation under these conditions. Taken together, we conclude that while PKGIα can be readily activated by oxidation *in vitro*, there is currently no direct evidence of oxidation-induced PKGIα activation *in vivo*.

The type I cGMP-dependent protein kinases (PKGI) play important roles in diverse physiological and pathophysiological processes. Their most studied and best understood signaling functions are in the cardiovascular system, where they control cardiac myocyte and smooth muscle contractility, but they also play key roles in synaptic plasticity, bone regulation, and beige/brown fat differentiation (1, 2, 3). As a result of differential splicing, mammalian cells express two PKGI isoforms, PKGIα and PKGIβ, which have unique N-terminal leucine zipper and autoinhibitory domains, but identical cyclic-nucleotide binding and catalytic domains (4, 5). The unique N-terminal domains cause PKGIα and PKGIβ to form homodimers, target the kinases to different substrates, and cause PKGIα to have a higher affinity for cGMP than PKGIβ (6, 7). The higher cGMP affinity in PKGIα correlates with a lower activation constant (*K*_a_) for cGMP (6).

While the PKGI enzymes are canonically activated downstream of nitric oxide– and natriuretic peptide–induced cGMP generation, various groups have reported oxidation-induced direct activation of the kinase (3, 4, 5, 6, 7). The first report was by Landgraf *et al.* (8), where the authors demonstrated that PKGIα was activated by oxidation in the presence of various metal ions. Using tryptic digests and mass spectrometry, they identified Cys^118^, Cys^196^, Cys^313^, and Cys^519^ as the cysteines most likely mediating this effect. In 2007, Bugoyne *et al.* (9) reported that PKGIα could be activated by hydrogen peroxide (H_2_O_2_)-induced disulfide formation between two cysteines at position 43 located at the C-terminal end of the leucine zipper/dimerization domain. However, we subsequently used cell-based and *in vitro* kinase assays to demonstrate that disulfide formation at Cys^43^ does not lead to PKGIα activation (10). We also found that the C43S mutation, which was generated to produce a “redox-dead” PKGIα, caused PKGIα to have an approximately five-fold lower sensitivity to cGMP-induced activation *in vitro*, compared to the WT enzyme (10). Our results were confirmed by Sheehe *et al.* (11). In addition, Sheehe *et al.* (11) concluded that H_2_O_2_-induced PKGIα activation was due to conversion of Cys^118^ to sulfonic acid and proposed that the negatively charged sulfonic acid interacted with basic residues distal to the autoinhibitory sequence.

During our previous studies, we found that cGMP-independent basal activity of purified Flag-epitope–tagged PKGIα increased after overnight storage in PBS with 100 ng/ml Flag peptide (Fig. 1). This activation occurred without the addition of an oxidizing agent and was associated with increased Cys^43^ crosslinking between the two peptide chains; however, while addition of DTT to the preactivated enzyme reversed Cys^43^ crosslinking, it did not reverse the increase in basal activity. The following studies were performed to probe the mechanism of PKGIα activation, under these conditions, and to assess whether this activation mechanism is physiologically important.

**Figure 1:**
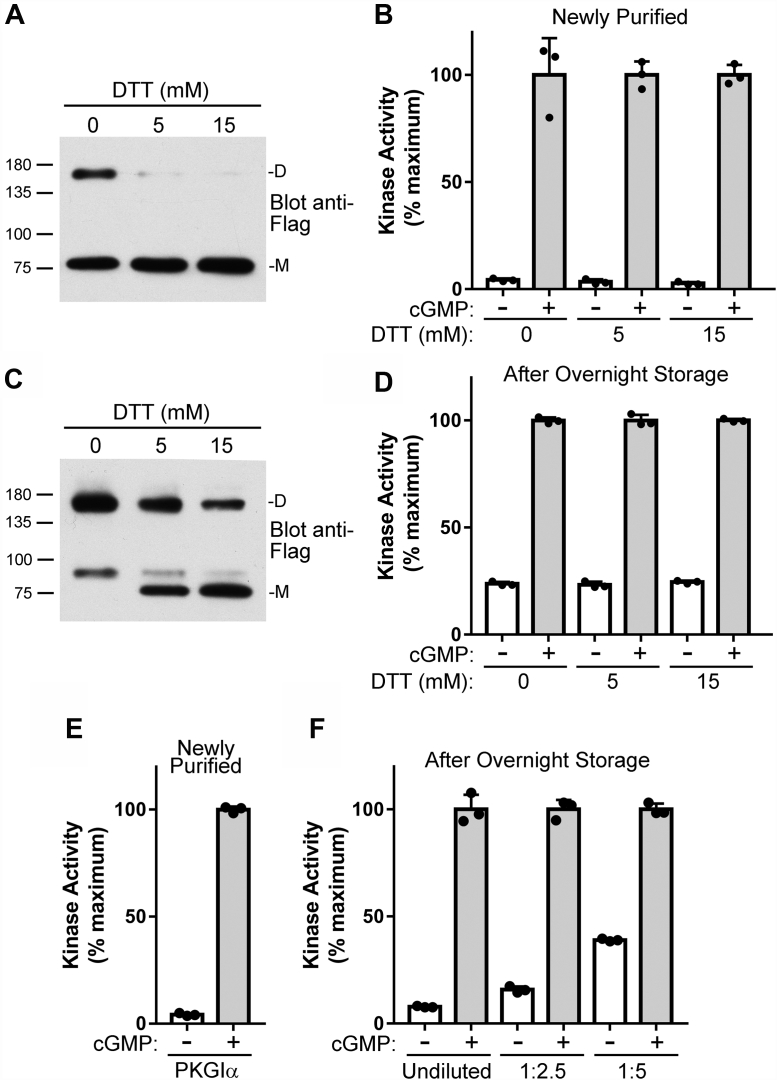
**PKGIα basal activity increases after overnight storage in Flag elution buffer.***A*, newly purified PKGIα was incubated for 1 h on ice in KPE buffer with the indicated amount of DTT and the level of Cys^43^-crosslinked PKGIα was determined by Western blotting (M = monomer, D = crosslinked dimer). *B*, kinase activity in the absence and presence of 10 μM cGMP was measured shortly after purification using an *in vitro* assay. *C* and *D*, the purified PKGIα was stored overnight at 4 °C in elution buffer and then incubated for 1 h with the indicated amounts of DTT in KPE buffer. The amount of crosslinked PKGIα with Cys^43^ oxidized was determined by Western Blotting (*C*) and kinase activity was measured (*D*). *E* and *F*, *in vitro* kinase activity of newly purified PKGIα (*E*) and after overnight storage with different levels of dilution in PBS (*F*). The figure shows data from a single protein preparation with assays performed in triplicate. Similar results were observed with two independent protein preparations. PKGI, Type I cGMP-dependent protein kinase.

## Results

### PKGIα basal activity increases after overnight storage in Flag elution buffer

Freshly prepared Flag-tagged PKGIα was diluted in KPE [10 mM potassium phosphate and 1 mM EDTA (pH 7.0)] buffer alone or KPE with 5 or 15 mM DTT. Immediately before performing activity assays, samples of the diluted kinases were added to SDS sample buffer containing 100 mM maleimide, and the amount of Cys^43^-crosslinked PKGIα was determined by nonreducing SDS-PAGE (Fig. 1*A*). The kinase was approximately 42% crosslinked in the absence of DTT and the crosslinking was almost completely reversed by DTT. We measured kinase activity on the diluted samples and found that, compared to the maximum cGMP-stimulated activity, basal activity was 4.3 ± 0.69% in the absence of DTT and 3.4 ± 1.2% or 2.7 ± 0.69% when incubated with 5 or 15 mM DTT, respectively (Fig. 1*B*). The slightly lower basal activity in the presence of DTT is similar to our previous results (10). The purified PKGIα was then stored at 4 °C overnight in elution buffer (PBS + 100 μg/ml Flag peptide). The next day, aliquots of the kinase were diluted in KPE buffer, with and without DTT, and kept on ice for 1 h. Western blots demonstrated that PKGIα diluted in KPE in the absence of DTT was completely oxidized with ∼75% migrating as a crosslinked dimer and ∼25% running as an unknown oxidation product at a higher apparent molecular weight than the reduced monomeric protein (Fig. 1*C*). In the presence of 5 or 15 mM DTT, both oxidation products were reduced to ∼40% or ∼60% monomeric/reduced, respectively. The basal kinase activity was increased to a similar extent under all three conditions (Fig. 1*D*). These results are consistent with our previous finding that PKGIα activity is independent of Cys^43^ crosslinking but demonstrates that the kinase is activated by some modification that is not easily reversed with DTT. Importantly, this modification occurred without adding H_2_O_2_ or other oxidizing agents to the purified protein (a second experiment with similar results is shown in Fig. S1).

It should be noted that the increase in basal kinase activity after overnight storage varied between different kinase preparations. This difference may be in part due to variable amounts of PKGIα in each preparation and thus the ratio of protein to buffer during storage. To test this hypothesis, we purified PKGIα and stored it overnight undiluted or diluted in elution buffer. As seen in Figure 1, *E* and *F*, the basal activity of a fresh PKGIα preparation was 4.7 ± 0.80% and increased to 7.8 ± 0.32% after overnight storage when not diluted. However, when aliquots of this preparation were diluted to 2- and 5-fold before storage, the basal activity increased to 16 ± 1.4% and 39 ± 0.57%, respectively. Importantly, adding 2-fold more Flag peptide to the elution buffer had no effect on the increased activity, indicating that activation was not being mediated by the peptide (data not shown). Therefore, given the variability in the level of PKGIα activation between protein preparations, all experiments in the main body of this article are from kinase reactions performed in triplicate on single protein preparations. To demonstrate qualitative reproducibility of the results, duplicate experiments using separate protein preparations are shown in Supplemental Data.

### PKGIα activation is prevented in the presence of reducing agents and metal chelators

**Figure 2:**
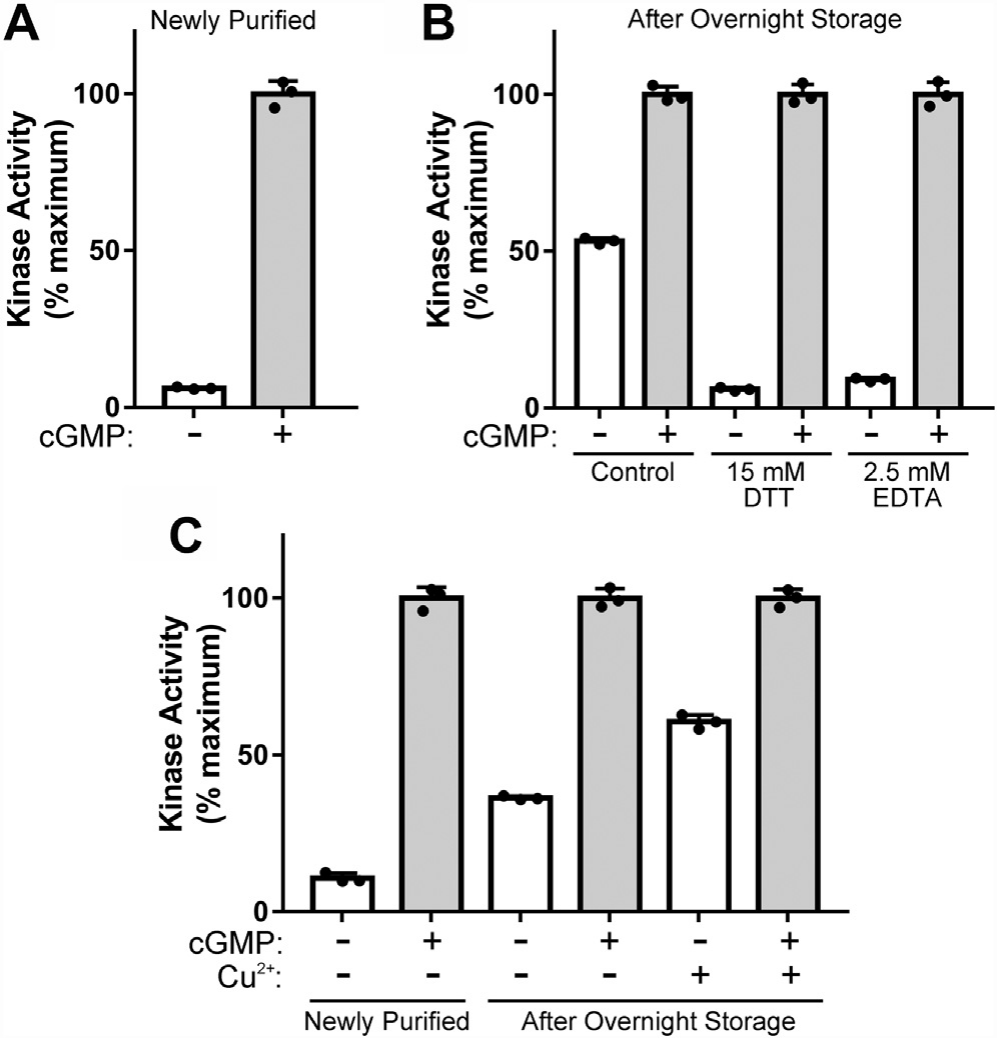
**PKGIα activation is prevented in the presence of reducing agents and metal chelators.***A*, *in vitro* kinase activity of PKGIα within 1 h of purification. *B*, PKGIα activity after overnight incubation in elution buffer alone or in elution buffer with the addition of DTT or EDTA, as indicated. *C*, PKGIα activity when freshly prepared and after overnight storage ±200 pM CuCl_2_. The figure shows data from a single protein preparation with assays performed in triplicate. Similar results were observed in two separate experiments. PKGI, Type I cGMP-dependent protein kinase.

Since short-term incubation with DTT did not reverse the kinase activation that had occurred during overnight storage, and metals have been shown to activate PKGIα, we assessed whether activation could be prevented by adding either DTT or the metal chelator EDTA before overnight storage. As seen in Figure 2*A*, the basal activity of newly purified PKGIα was 6.2 ± 0.34% and increased to 53 ± 0.97% after overnight storage in elution buffer alone, but in samples stored in elution buffer with DTT or EDTA, the increase in basal activity was largely prevented (6.0 ± 0.53 and 9.1 ± 0.58%, respectively) (Fig. 2*B*). To directly test the effect of heavy metals, we measured the basal activity of newly purified PKGIα and then stored it overnight with and without added Cu^2+^. Basal activity of newly prepared kinase was 11 ± 1.6% of maximum and increased to 36 ± 0.62% *versus* 61 ± 2.2% after overnight storage in the absence or presence of added Cu^2+^, respectively (Fig. 2*C*). Taken together, these results are consistent with oxidation-induced activation being driven by the presence of trace metals in the storage buffer.

### PKGIα activation is independent of Cys^43^ oxidation

**Figure 3:**
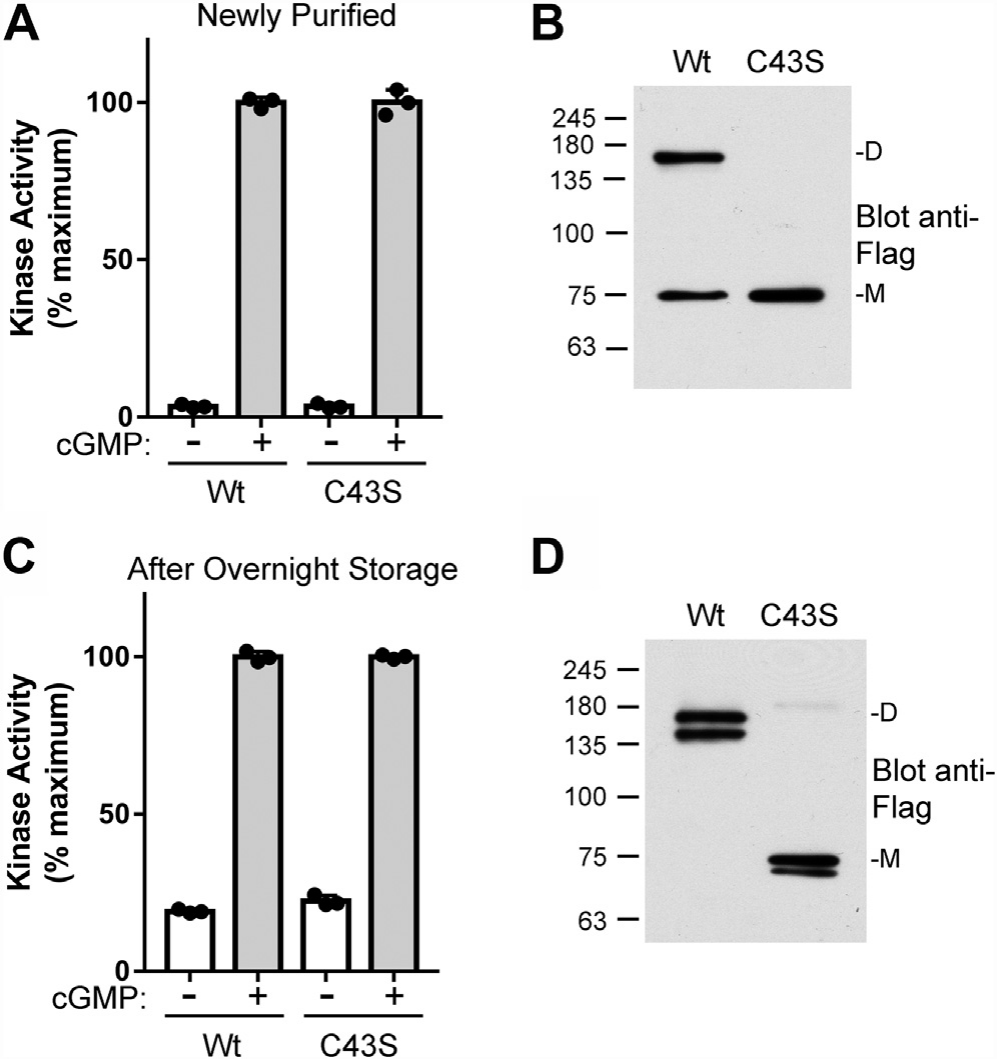
**PKGIα activation is independent of C43S oxidation.***A*, the activities of freshly purified WT and C43S PKGIα were analyzed by *in vitro* kinase assays. *B*, Western blotting showing amount of Cys^43^-crosslinked PKGIα in the two preparations immediately after purification (M = monomer, D = crosslinked dimer). *C*, *in vitro* kinase assays performed using the protein preparations from (*A*) after overnight storage in elution buffer. *D*, Western blot showing the amount of crosslinked PKGIα after overnight storage. The figure shows data from a single protein preparation with assays performed in triplicate. Similar results were observed with two independent protein preparations. PKGI, Type I cGMP-dependent protein kinase.

Even though Cys^43^ crosslinking was not directly responsible for PKGIα activation, it is still possible that Cys^43^ plays role in the observed activation. Therefore, we compared activation between WT and C43S PKGIα. The basal activity of newly purified WT and C43S PKGIα were 4.6 ± 1.8 and 6.1 ± 3.2%, respectively (Fig. 3*A*). The amount of crosslinked WT PKGIα was ∼49% and as expected, no crosslinking was seen in the C43S mutant (Fig. 3*B*). After overnight storage, basal activity of WT and C43S PKGIα increased to a similar extent, 33 ± 0.91 and 31 ± 0.50% of maximum activity, respectively (Fig. 3*C*). Similar results are shown in Fig. S3. The WT enzyme was completely crosslinked at Cys^43^ (Fig. 3*D*); however, it should be noted that the crosslinked WT and the monomeric C43S PKGIα bands appeared as doublets, suggesting that oxidation events beyond Cys^43^ crosslinking were occurring. Similar doublets have been reported by Donzelli *et al.* (12) and are thought to be the result of disulfide bond formation between Cys^118^ and C^196^.

Prysyazhna *et al.* (13) reported that Cys^43^ crosslinking alters PKGIα's activation by cGMP; however, in a previous study, we found that Cys^43^ crosslinking had no effect on the *K*_a_ for cGMP (10). Other noncanonical cyclic nucleotides have been reported to activate PKGIα (14, 15), and it is possible that Cys^43^ crosslinking could alter the affinity for these nucleotides. To test this possibility, we performed kinase reactions with increasing concentrations of cAMP, cCMP, and cIMP using oxidized and reduced PKGIα (Fig. S4). We found that Cys^43^ crosslinking had no effect on the *K*_a_ for any of these nucleotides.

### Mutation of either Cys^118^ or Cys^196^ reduces oxidation-mediated PKGIα activation

**Figure 4:**
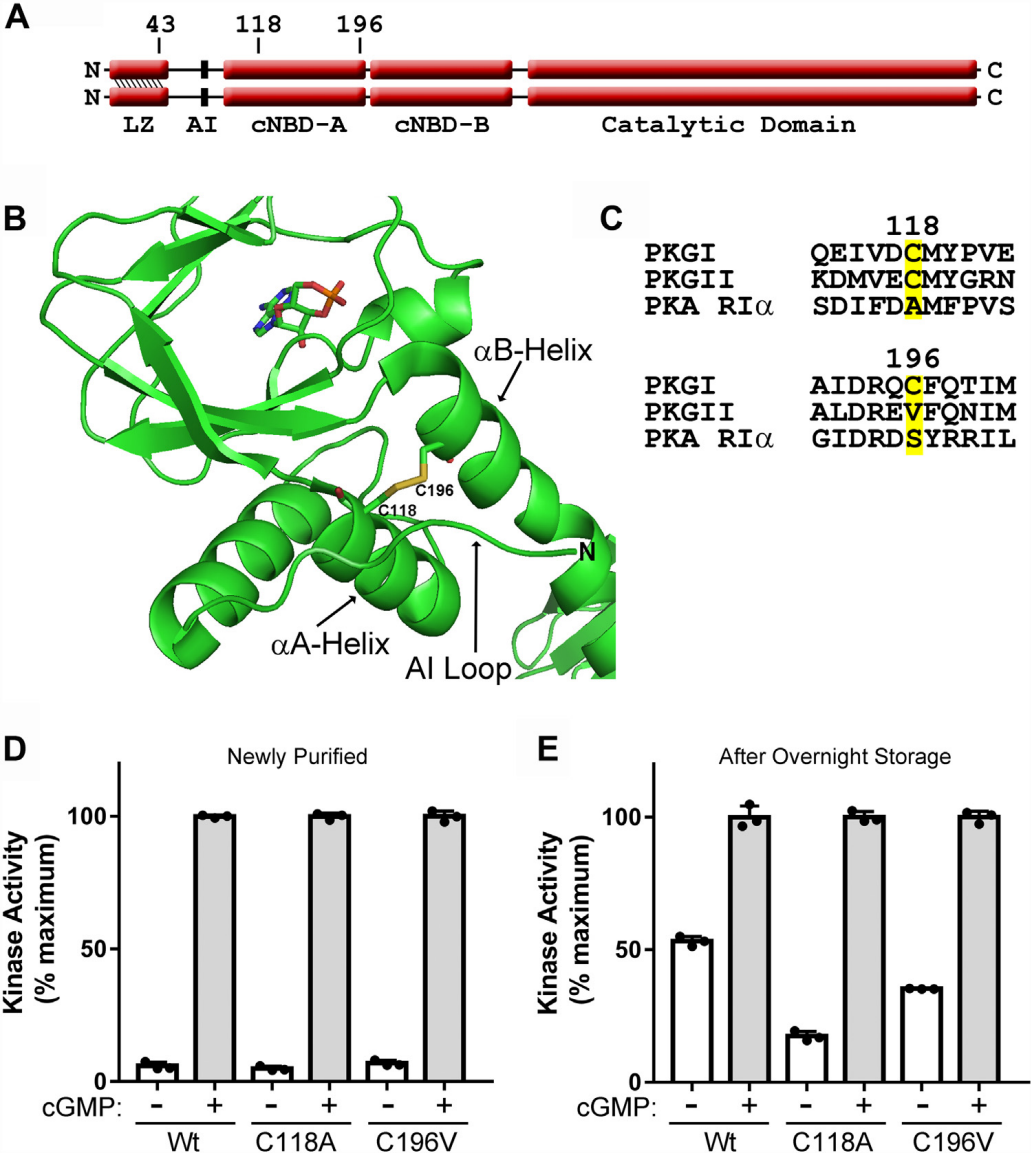
**Mutation of either Cys**^**118**^**or Cys**^**196**^**reduces oxidation mediated PKGIα activation.***A*, domain map of PKGIα showing the location of Cys^43^, Cys^118^, and Cys^196^. *B*, structure of PKGIα showing the location of the Cys^118^/Cys^196^ disulfide bond (PDB: 3SHR). *C*, sequence alignment between PKGIα with the homologous sequences in PKGII and PKA RIα. *D*, kinase assays performed on WT, C118A, and C196V PKGIα immediately after purification. *E*, kinase assays performed after overnight storage. The figure shows data from a single protein preparation with assays performed in triplicate. Similar results were observed with two independent enzyme purifications. AI, autoinhibitory loop; cNBD-A and cNBD-B, the two cyclic nucleotide binding domains; LZ, leucine zipper; PKGI, Type I cGMP-dependent protein kinase.

In order to determine if oxidation of PKGIα Cys^118^ or Cys^196^ was responsible for the overnight activation, we used mutagenesis to change the cysteines to nonoxidizable residues. These residues are located in the first cGMP-binding pocket (Fig. 4*A*), and a disulfide bond was seen between these residues in a crystal structure of the isolated CNB-A/B domains [Fig. 4*B* and (16)]. Since we wanted to produce mutations that prevent oxidation-induced activation, but otherwise have no effect on basal kinase activity or cGMP response, we identified amino acid differences at these positions in homologous proteins, reasoning that changing the cysteines to these residues would be less likely to disrupt folding of the cGMP-binding pocket. Thus, we aligned PKGI, PKGII, and PKA RIα amino acid sequences and found that RIα has an alanine at the position analogous to Cys^118^ and that PKGII has a valine and RIα has a serine at the position analogous to Cys^196^ (Fig. 4*C*). Thus, we compared activation of WT, C118A, and C196V PKGIα. As seen in Figure 4*D*, the basal activities of WT, C118A, and C196V were 5.9 ± 1.4, 5.0 ± 0.9, and 6.9 ± 1.1% of maximum, respectively. The next day, basal activities increased to 53 ± 1.9 of maximum for WT but only to 17 ± 1.8 and 35 ± 0.1% for the C118A and C196V mutant kinases, respectively (Fig. 4*E*). Similar results for a separate enzyme purification are shown in Fig. S5. While mutation of Cys^118^ had the most pronounced effect on preventing activation, the C196V mutation also reduced the level of activation. Together, these data demonstrate that in addition to oxidation of Cys^118^, oxidation of Cys^196^ and/or other residues can also induce PKGIα activation.

### PKGIβ is not highly activated by overnight oxidation

**Figure 5:**
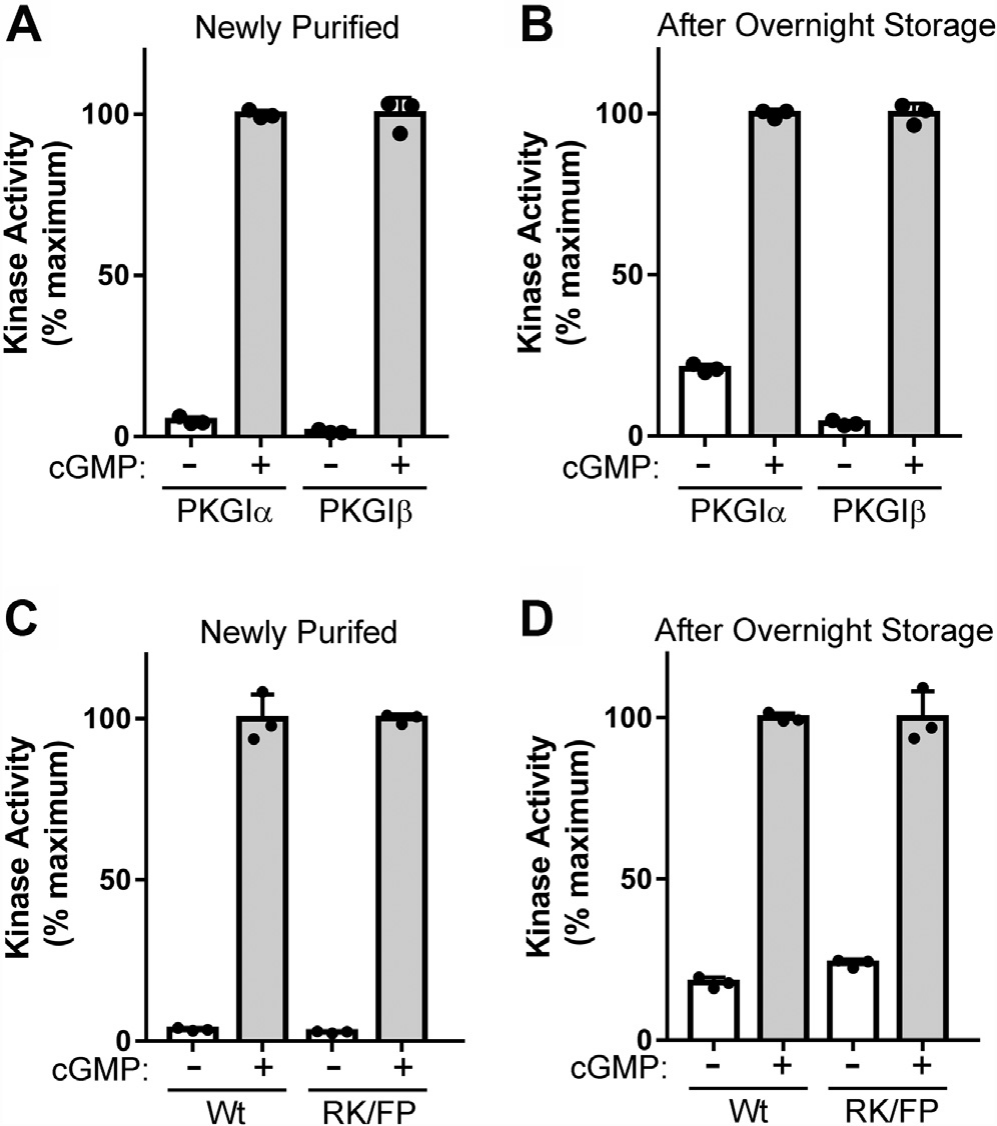
**PKGIβ is not highly activated during overnight storage.***A*, kinase assays performed with purified PKGIα and PKGIβ immediately after purification. *B*, kinase assays performed after overnight storage in elution buffer. *C*, kinase assays performed with WT and R82F/K83P (RK/FP) PKGIα within 1 h of purification. *D*, kinase assays performed on the protein preparations shown in *C* after 20-h storage in elution buffer at 4 °C. The figure shows data from a single protein preparation with assays performed in triplicate. Similar results were observed with two independent enzyme purifications. PKGI, Type I cGMP-dependent protein kinase.

Since PKGIα and PKGIβ have identical sequences in their first cyclic nucleotide-binding pockets (which contain both Cys^118^/Cys^133^ and Cys^196^/Cys^311^), we examined whether PKGIβ is also activated during overnight storage. PKGIα and PKGIβ purified and immediately assayed showed a basal activity of 4.9 ± 1.2% and 1.6 ± 0.54% of maximum, respectively (Fig. 5*A*). After overnight storage at 4 °C, as expected, the basal activity of PKGIα increased to 21 ± 1.3%, whereas the basal activity of PKGIβ only slightly increased to 4.0 ± 0.78%. Similar results are shown in Fig. S6, *A* and *B*. These findings are consistent with those reported by Sheehe *et al.* (11), who showed that unlike PKGIα, purified PKGIβ was resistant to H_2_O_2_-induced activation.

### Testing the activation mechanism proposed by Sheehe *et al.*

To explain the different response of PKGIα and PKGIβ to H_2_O_2_-induced activation, Sheehe *et al.* (11) proposed a mechanism in which basic residues unique to the PKGIα autoinhibitory loop interacted with a negatively charged sulfonic acid moiety formed at Cys^118^ in response to H_2_O_2_. We tested this mechanism by mutating the basic residues found in the PKGIα autoinhibitory loop to the corresponding nonbasic residues in PKGIβ. Specifically, we simultaneously mutated PKGIα Arg^82^ to Phe (R82F) and Lys^83^ to Pro (K83P). We found that in freshly purified preparations, the basal activity of the mutant protein (referred to as RK/FP) was similar to WT PKGIα, and that the mutations did not prevent activation after overnight storage (Fig. 5, *C* and *D*). Similar results are shown in Fig. S6, *C* and *D*. These results are not consistent with the activation mechanism proposed by Sheehe *et al.*, but suggest different mechanisms, tested below.

### Residues throughout the PKGIα autoinhibitory region mediate oxidant-induced activation of PKGIα

**Figure 6:**
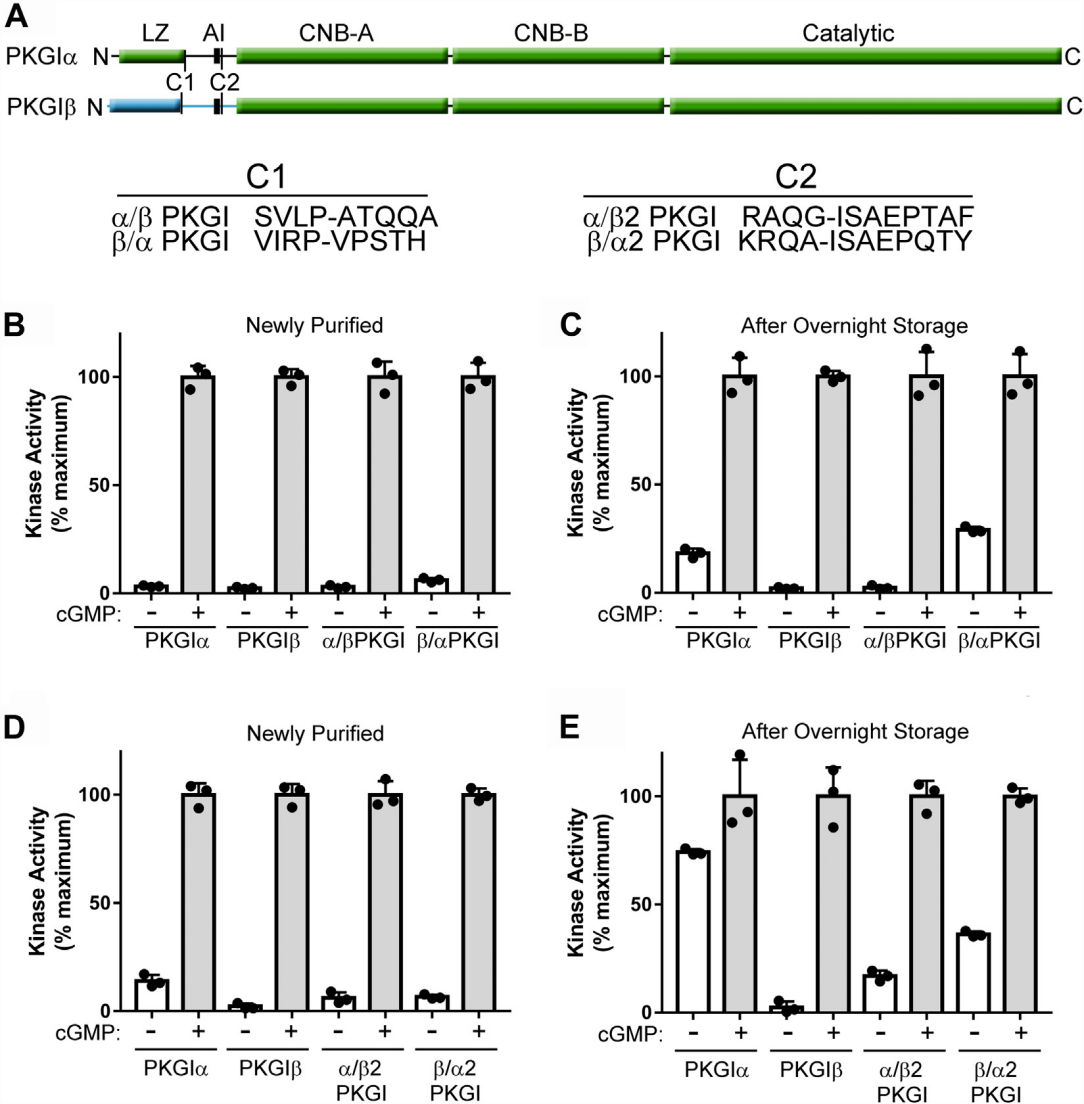
**Residues throughout the PKGIα autoinhibitory region mediate oxidant-induced activation of PKGIα.***A*, domain maps of PKGIα and PKGIβ showing the locations of the chimeric splice sites (C1 and C2). The amino acid sequences of the chimeric proteins are shown below, with the splice sites indicated by *hyphens*. The location of the pseudosubstrate sequence is indicated by a *black box*. *B*, kinase assays performed on WT and leucine zipper swapped C1 chimeric PKGI within 1 h of purification (α = PKGIα, β = PKGIβ, α/β = chimeric protein with PKGIα leucine zipper and PKGIβ autoinhibitory loop, β/α = chimeric protein with PKGIβ leucine zipper and PKGIα autoinhibitory loop). *C*, kinase assays performed using the protein preparations shown in panel *B* after 20-h storage in elution buffer at 4 °C. *D*, kinase assays performed on WT and C2 chimeric PKGI within 1 h of purification (α = PKGIα, β = PKGIβ, α/β2 = chimeric PKGI with PKGIα residues N-terminal to the splice site, β/α2 = chimeric protein with PKGIβ residues N-terminal to the splice site). *E*, kinase assays performed using the protein preparations shown in panel *D* after 20-h storage in elution buffer at 4 °C. AI, autoinhibitory loop; CNB-A/B, cyclic nucleotide binding domains; LZ, leucine zipper; PKGI, Type I cGMP-dependent protein kinase.

Since overnight storage differentially affected the basal activities of PKGIα and PKGIβ, we made chimeric enzymes in which we swapped the leucine zipper domains between the two kinases (chimera C1, Fig. 6*A*). The α/β kinase had a PKGIα leucine zipper with a PKGIβ autoinhibitory domain and the β/α kinase had the opposite (the remaining sequences are identical between the two isoforms). We then performed *in vitro* kinase assays on freshly purified PKGIα, PKGIβ, PKGIα/β, and PKGIβ/α and found that they had similar basal activities (Figs. 6*B* and S7*A*). After overnight storage, the basal activities of PKGIα and PKGIβ/α increased to a similar degree, but the basal activities of PKGIβ and PKGIα/β remained low (Figs. 6*C* and S7*B*). Thus, activation required residues in the PKGIα autoinhibitory domain. To localize the residues responsible for activation, we made another set of complementary chimeric enzymes by swapping the amino acids N-terminal to the ISAEP amino acid sequence, which is conserved in both isoforms and located after the pseudosubstrate sequence in the autoinhibitory domain (chimera C2, Fig. 1*A*). After overnight storage, basal activity increased in both chimeric enzymes, but the increase was less than that seen for WT PKGIα (Fig. 6, *D* and *E*). The same pattern of activation was seen with separate enzyme preparations (Fig. S7, *C* and *D*), suggesting that activation is most likely mediated through an additive effect involving residues throughout the PKGIα autoinhibitory loop.

### Testing the effect of acidic residue mutations at PKGIα Cys^118^ and PKGIβ Cys^196^ on kinase activity

**Figure 7:**
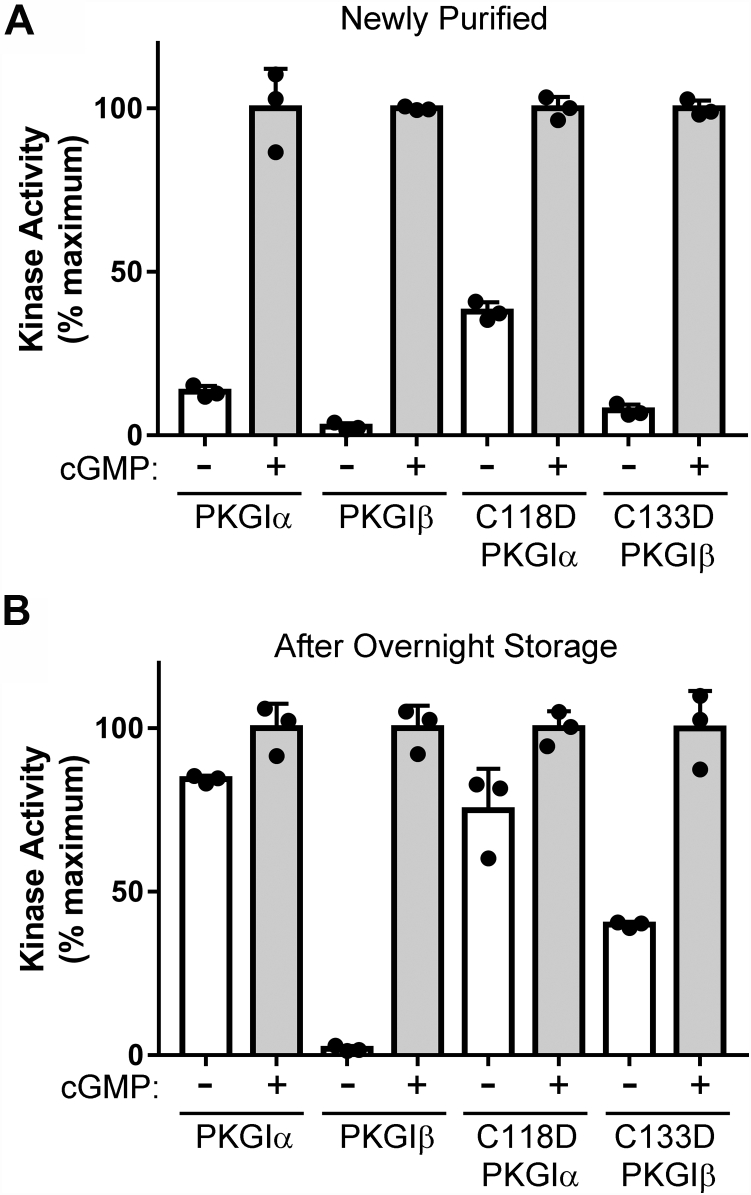
**Testing the effect of acidic residue mutations at PKGIα Cys**^**118**^**and PKGIβ Cys**^**196**^**on kinase activity.***A*, kinase assays using newly purified PKGIα, PKGIβ, C118D PKGIα, and C133D PKGIβ. Assays were performed within 1 h of purification. *B*, kinase assays performed on the protein preparations shown in A after 20-h storage in elution buffer at 4 °C. The figure shows data from a single protein preparation with assays performed in triplicate. Similar results were observed with two independent enzyme purifications. PKGI, Type I cGMP-dependent protein kinase.

Since Sheehe *et al.* demonstrated that H_2_O_2_ treatment caused conversion of Cys^118^ to a negatively charged acid moiety which then induces kinase activation, we examined the effect of mutating Cys^118^ to Asp. We also assessed the corresponding mutation in PKGIβ (*i.e.*, C133D). Freshly purified C118D PKGIα and C133D PKGIβ had higher basal activities than the WT enzymes (Fig. 7*A*). The basal activities of both mutants further increased after overnight storage (Fig. 7*B*), indicating that the enzymes were activated by modification of one or more additional site(s). Separate enzyme preparations with similar results are shown in Fig. S8.

### H_2_O_2_ does not activate PKGIα in cultured cells

**Figure 8:**
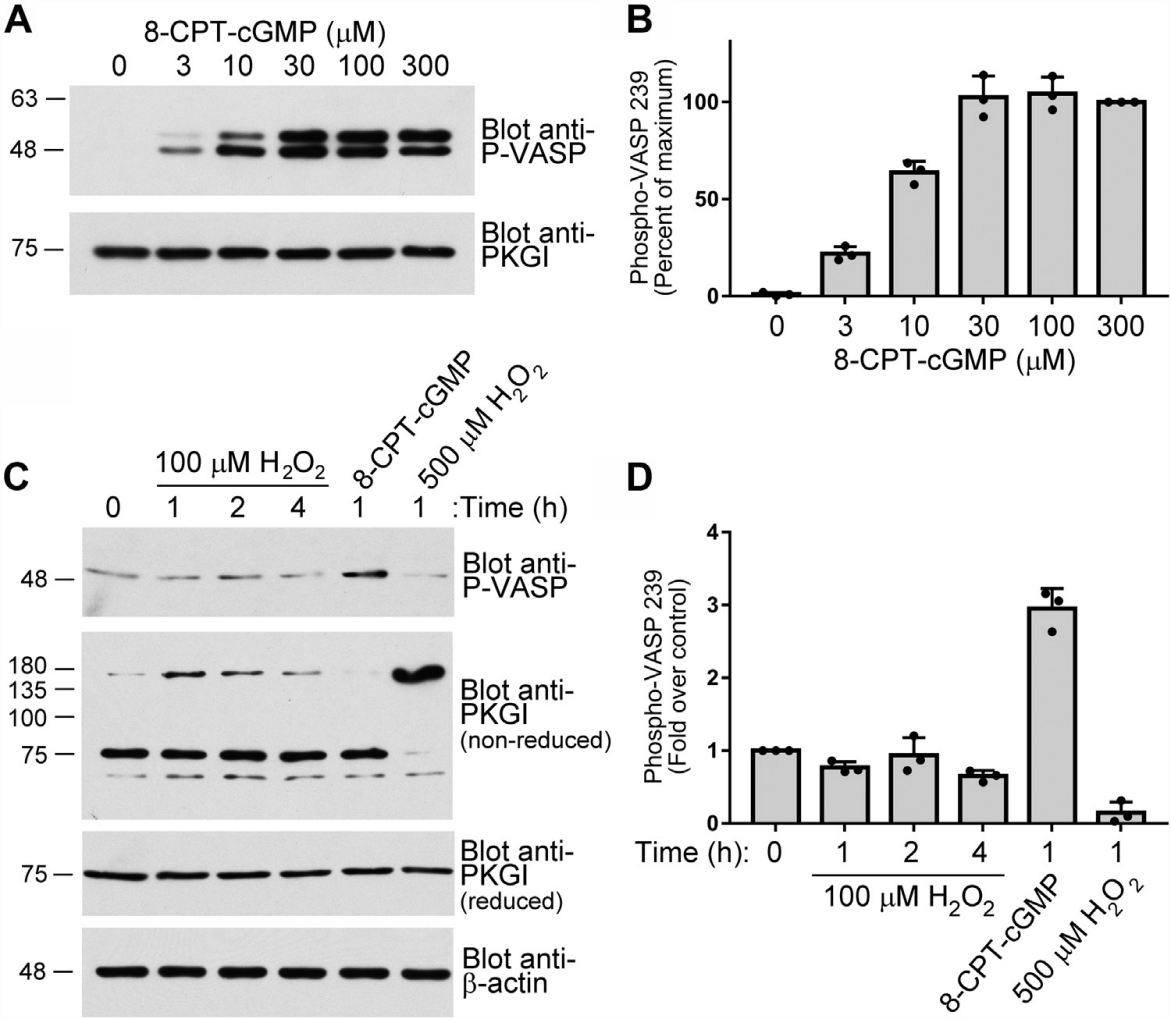
**H**_**2**_**O**_**2**_**does not activate PKGIα in H9c2 cells.***A*, H9c2 cells were treated with the indicated concentrations of 8-CPT-cGMP for 1 h. The amount of VASP phosphorylated on serine 239 was analyzed by immunoblotting using an antibody specific for pVASP (phospho-Ser^239^) (*upper panel*) and equal loading was determined by blotting for PKGI under reducing conditions (*lower panel*). *B*, quantification of three independent experiments as described in (*A*). *C*, H9c2 cells were treated with 100 μM H_2_O_2_ for the indicated times or treated with 3 μM 8-CPT-cGMP or 500 μM H_2_O_2_ for 1 h. In addition to VASP phosphorylation on Ser^239^ (*top panel*), Cys^43^-mediated crosslinking of PKGIα was assessed under nonreducing conditions (*second panel*), and total PKGIα was assessed under reducing conditions (*third panel*); β-actin served as a loading control (*bottom panel*). *D*, quantification of pVASP (phospho-Ser^239^) from three independent experiments as described in (*C*). The amounts of phospho-Ser^239^ VASP and β-Actin were determined by immunoblotting and densiometric scanning using Image J. H_2_O_2_, hydrogen peroxide; PKGI, Type I cGMP-dependent protein kinase; VASP, vasodilator-stimulated phosphoprotein.

The H9c2 cell line was originally derived from embryonic rat heart (17). The cell line expresses endogenous PKGIα and vasodilator-stimulated phosphoprotein (VASP), a well characterized PKGI substrate. To assess how H9c2 cells respond to cGMP-induced PKGIα activation, we treated the cells with increasing amounts of 8-pCPT-cGMP and monitored VASP Ser239 phosphorylation. We found that the level of VASP phosphorylation reached ∼25% of maximum at 3 μM and peaked at 30 μM 8-pCPT-cGMP (Fig. 8, *A* and *B*). Next, we treated cells with 100 μM H_2_O_2_ for 1, 2, or 4 h or 500 μM H_2_O_2_ for 1 h (at longer time points with 500 μM H_2_O_2_, the cells started to detach from the plate). In parallel, cells were treated with 3 μM 8-pCPT-cGMP for 1 h, which induced a three-fold increase in VASP phosphorylation (Fig. 8, *C* and *D*). While 100 μM H_2_O_2_ increased the amount of Cys^43^-crosslinked PKGIα (indicating PKGIα oxidation, as determined by nonreducing gel electrophoresis, second blot in Fig. 8*C*), it did not lead to increased VASP phosphorylation (top blot in Fig. 8*C*, with three independent experiments quantified in Fig. 8*D*). In the presence of 500 μM H_2_O_2_, almost all PKGIα is crosslinked and the level of VASP phosphorylation actually deceases (Fig. 8*D*). Similar results were seen in mouse myoblast C2C12 cells (Fig. S9). Taken together, these data demonstrate that even under robust oxidative conditions, which result in a high level of oxidant-induced PKGIα Cys^43^ crosslinking, PKGIα is not activated in H9c2 or C2C12 cells.

## Discussion

**Figure 9:**
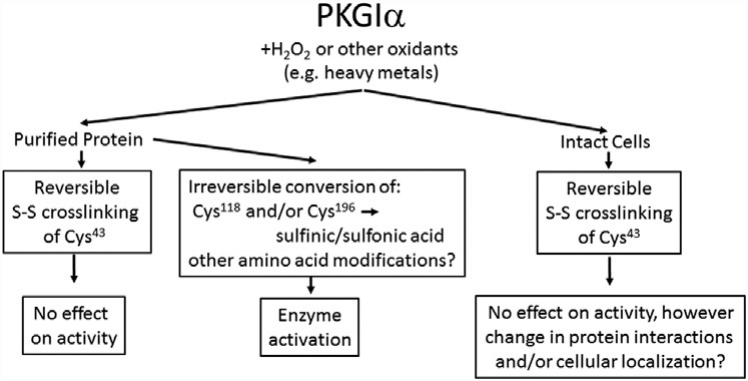
**Schema showing different ways oxidation may affect purified PKGIα *versus* PKGIα signaling in intact cells.** Oxidant-induced reversible Cys^43^ crosslinking of purified PKGIα has no effect on kinase activity. However, oxidant- and metal-induced irreversible conversion of Cys^118^ or Cys^196^ to an acidic moiety (and modification of other amino acids) leads to kinase activation. In intact cells (or possibly *in vivo*), oxidant-induced crosslinking of Cys^43^ may change cellular PKGIα targeting; however, oxidant-induced modification of Cys^118^ (or of other amino acids) has not been demonstrated. PKGI, Type I cGMP-dependent protein kinase.

PKGIs play key roles in the cardiovascular system and are the indirect targets of a number of pharmacological agents that work by raising intercellular cGMP levels (18). While a number of studies over the last 30 years have provided a wealth of insight into PKGI regulation and signaling, new findings continue to emerge. These findings include noncanonical modes of kinase regulation, detailed descriptions of mechanisms of cellular targeting and compartmentalization, and new downstream substrates which regulate novel signaling pathways or cellular processes. One of the most interesting areas of study has been the direct regulation of PKGIα activity by oxidation, which remains controversial (19, 20). In this article, we show that while PKGIα is activated by oxidation *in vitro*, oxidation does not directly activate the kinase in intact cells. A mechanistic schema for the different ways oxidation affects the activity of purified PKGIα *versus* PKGIα activity/signaling in intact cells is shown in Figure 9.

### Metal-induced activation of purified PKGIα *in vitro*

The first description of PKGI regulation by oxidation was reported by Landgraf *et al.*, who found that PKGI purified from bovine lung could be activated by incubation with various metals, including Ag^+^, Hg^+^, Cu^+^, Cu^2+^, and Fe^3+^. They also demonstrated that activation by Cu^2+^ was blocked by coincubation with the reducing agent DDT or the metal chelator EDTA (8). These results are consistent with our current findings, which suggest that trace metals in the buffers (or carried over from cell extracts during purification) induced PKGIα activation during storage. These authors found that Cu^2+^-induced activation could be reversed by removing the Cu^2+^ by gel filtration and reducing the enzyme with DTT, and they concluded that activation was due to the formation of intrachain disulfide bond(s) between either Cys^118^:Cys^196^ or Cys^313^:Cys^519^. Consistent with this conclusion, Donzelli *et al.* (12) proposed that PKGIα could be activated by nitroxyl-induced disulfide bond formation between Cys^118^ and Cys^196^, and Osborne *et al.* (16) observed a disulfide bond between these residues in a crystal structure of the PKGIα cyclic nucleotide-binding domains. In contrast to activation being induced by formation of a Cys^118^:Cys^196^ disulfide bond, Shehee *et al.* found that H_2_O_2_-induced oxidation converted Cys^118^ to sulfonic acid and proposed that PKGIα activation was caused by interaction between the newly formed acidic moiety and basic residues unique to the PKGIα autoinhibitory domain. While our current results are consistent with the conversion of Cys^118^ to sulfonic acid, we found that mutation of the basic residues that were predicted to interact with the sulfonic acid moiety did not prevent PKGIα activation.

### Oxidant-induced PKGIα crosslinking at Cys^43^ does not increase kinase activity but may alter cellular targeting

In 2007, Burgoyne *et al.* (9) reported that PKGIα could be activated by oxidant-induced disulfide formation between two cysteines at position 43 located at the end of the leucine zipper in each PKGIα peptide in the homodimer. A knock-in mouse containing PKGIα with a C43S mutation has a phenotype consistent with loss of PKGIα function, which implied oxidation-induced PKGIα activation was an important physiological mechanism for regulation the kinase (21, 22, 23, 24). However, we and others have reported that Cys^43^ crosslinking does not increase PKGIα kinase activity *in vitro* (10, 11). Importantly, we found that the ‘redox-dead” C43S mutation caused PKGIα to be 5-fold less sensitive to cGMP-induced activation (10). A decrease in cGMP sensitivity for C43S PKGIα was also seen by Shehee *et al.* (11). The reduced cGMP affinity could theoretically explain the loss-of-function phenotype of the C43S PKGIα knock-in mouse.

The PKGIα leucine zipper domain is involved in mediating homodimerization of the enzyme and also targets the kinase to specific substrates (25, 26, 27). The importance for proper PKGIα targeting *in vivo* has been demonstrated by a knock-in mouse with mutations in the leucine zipper that prevent dimerization. These mice show adult onset hypertension and are more sensitive to cardiac pressure overload than wild-type littermates (*i.e.*, increased hypertrophy, systolic/diastolic dysfunction, and mortality) (28, 29). While PKGIα is dimeric in the absence of Cys^43^ crosslinking, crosslinking may stabilize the helical conformation of the leucine zipper, especially at its C-terminus, and may confine the conformation of an interface for protein–protein interactions (30). Consistent with this, Cys^43^ crosslinking increases the interaction between PKGIα and two of its known interacting proteins, MYPT1 and RhoA, *in vitro* (9).

### H_2_O_2_ does not activate PKGIα in cardiac myocyte-derived H9c2 cells or C2C12 myoblasts

The H9c2 cell line derived from embryonic rat hearts has been used as an alternative to primary cardiac myocytes (17). The cell line expresses PKGIα and VASP and thus serves as an ideal platform to study PKGIα signaling in a cellular context. VASP phosphorylation is a sensitive readout for PKGIα activation, and treating these cells with cell-permeable cGMP analogs leads to robust VASP phosphorylation. However, we were unable to detect VASP phosphorylation after treatment with relatively high amounts of H_2_O_2_ in either H9c2 or C2C12 cells. While H_2_O_2_ is an endogenous signaling molecule, the amounts found *in vivo* are thought to normally be in the low μM range but may reach higher levels under pathophysiological conditions (31). The finding that oxidant-induced PKGIα activation *in vitro* is due to irreversible modification of cysteines to sulfinic and/or sulfonic acids strongly argues against it serving as a dynamic signaling mechanism *in vivo*.

### H_2_O_2_ may increase PKGIα substrate phosphorylation by activating soluble guanylate cyclase or inhibiting phosphatases

If PKGIα is not activated by oxidation in intact cells, how are we to account for experiments showing that tissues from C43S knock-in mice are resistant to H_2_O_2_ induced relaxation, but still relax in response to cGMP-analogs and nitro vasodilators? Previous studies have shown that treatment with H_2_O_2_ can activate soluble guanylate cyclase (sGC) (32, 33, 34). This activation seems to require a reaction between H_2_O_2_ and superoxide to form hydroxyl radicals (32) or metabolism of H_2_O_2_ by catalase to form Compound I (33). However, sGC can also be inhibited by oxidation (35). Thus, treatment with H_2_O_2_ may transiently activate sGC and produce a localized pool of cGMP. In this case, relaxation would rely on properly localized PKGIα with a high sensitivity to cGMP which can respond to this pool. Under such conditions, the loss of cGMP affinity and/or mislocalization of C43S PKGIα could explain the failure of tissues from the knock-in mouse to relax in response to H_2_O_2_. It should be noted that H9c2 and C2C12 cells do not express sGC, since PKGIα is not activated in response to nitric oxide donors (data not shown).

An apparent increase in PKGIα activity may also be due to inhibition of serine/threonine phosphatases by H_2_O_2_. Humphries *et al.* (36) found that enhanced cAMP-dependent protein kinase (PKA) substrate phosphorylation, seen when HeLa cells are treated with the sulfhydryl-specific oxidant diamide, is blunted in the presence of phosphatase inhibitors, indicating that the enhanced phosphorylation is due to phosphatase inhibition rather than kinase activation. While the exact phosphatases affected were not identified, PP1 and PP2A are known to dephosphorylate the PKA substrate CREB (37, 38), which is also a substrate for PKGI (39). Interestingly, Kim *et al.* (40) found that H_2_O_2_ treatment inhibits PP1 and PP2A in primary human diploid fibroblasts. Whether oxidant-induced phosphatase inhibition enhances PKGIα signaling in cells is currently unknown.

### Study limitations and future directions

A limitation of this study is that in assessing the ability of oxidants to activate PKGIα in cells, we only examined one substrate (VASP) in two cell lines (H9c2 and C2C12). To analyze phosphorylation of other direct PKGIα substrates, we have tested a number of phospho-specific antibodies, but we found that they are not sensitive enough to detect substrate phosphorylation at endogenous protein levels in these cells. We have examined a number of primary cells and established cell lines, but we were unable to identify cells in addition to H9c2 and C2C12 cells which contain sufficient amounts of PKGIα without expressing sGC. Another limitation of this study is that cell culture conditions may not reflect conditions found *in vivo*. It is possible that under certain pathophysiological conditions, which result in very high oxidant levels, PKGIα may become activated by oxidation-induced modification of Cys^118^ to an acid; but to our knowledge, there is no evidence that this modification occurs in cultured cells or *in vivo*. We are currently examining if Cys^43^ crosslinking changes PKGIα targeting in cells and the mechanism through which H_2_O_2_ may activate sGC.

## Conclusion

In conclusion, the physiological significance of oxidation-induced PKGIα activation is doubtful. This is based on three main findings: (i) the observed *in vitro* oxidation is driven by metals in the presence of atmospheric oxygen; (ii) the activating modification is not easily reversed, arguing against a dynamic regulatory mechanism; and (iii) even in the presence of higher than physiological H_2_O_2_ levels, oxidant-induced PKGIα activation is not observed in cultured cells.

## Experimental procedures

### Materials

Fetal bovine serum, horseradish peroxidase (HRP)-conjugated anti-Flag M2 antibody, anti-Flag M2 affinity gel, and Flag peptide were from Sigma. Phospho-VASP (Ser239) Antibody was from Cell Signaling Technology. HRP-conjugated goat anti-mouse (115-035-062) and goat anti-rabbit (111-035-046) antibodies were from Jackson Immuno Research. Kemptide was from AnaSpec, Inc. Cyclic nucleotide analogs were from BioLog Life Science Institute, and general laboratory reagents were from Fisher Scientific, Sigma Life Science, or Bio-Rad Laboratories.

### Vector constructs

Flag-tagged WT PKGIα, WT PKGIβ, and C43S PKGIα have been described previously (10). Additional mutations and chimeric PKGIα/PKGIβ were produced using overlapping extension PCR (41, 42). PCR products were digested with BamHI and XhoI and ligated into BamHI/XhoI cut pFlag-D (10). All constructs derived by a PCR step were sequenced.

### Cell culture and transfection

HEK293T/17 (ATCC ACS-4500), C2C12 (ATCC CRL-1772), and H9c2(2-1) (ATCC CRL-1446) cells were grown at 37 °C in a 5% CO_2_ atmosphere in Dulbecco's Modified Eagle Medium supplemented with 10% fetal bovine serum. Cells were transfected using Lipofectamine^2000^ (Life Technologies) according to the manufacturer’s instructions.

### Kinase purification

Flag-tagged WT and mutant PKGIα and PKGIβ were purified as described (10). Briefly, Flag-tagged expression vectors were transiently transfected into HEK293T cells and 24 h later, cells were lysed in buffer A [PBS, 0.1% NP40, and protease inhibitor cocktail (Calbiochem #539131)]. Lysates were cleared by centrifugation and incubated with anti-Flag beads for 1 h at 4 °C. Beads were extensively washed, and PKG was eluted in PBS with 100 μg/ml Flag peptide. Purified kinases were either used immediately or assayed after overnight storage at 4 °C in elution buffer (∼20 h). For some samples, kinases were diluted with an equal volume of PBS containing a two-fold concentration of added reagents (*i.e.*, 30 mM DTT, 5 mM EDTA, or 200 pM Cu^2+^) before overnight storage.

### *In vitro* kinase assays

Purified kinase was diluted to ∼1 ng/μl in KPEB Buffer [10 mM potassium phosphate (pH 7.0), 1 mM EDTA, and 0.1% bovine serum albumin]. For some reactions, KPEB contained the amount of DTT indicated in the text, and the diluted samples were kept on ice for 1 h before the kinase reactions were performed. Dose/response reactions for noncanonical cyclic nucleotides were performed as described (10), using increasing concentrations of the indicated cyclic nucleotides. Cyclic nucleotide *K*_a_ values were calculated and compared using GraphPad Prism 8. Reactions were initiated by adding 10 μl diluted kinase to 5 μl 3× kinase reaction mix [120 mM Hepes (pH 7.4), 30 mM MgCl_2_, 180 μM ATP, 180 μCi/ml [γ-^32^P] ATP, and 1.56 mg/ml Kemptide] with or without 30 μM cGMP. Kinase reactions were run for 1.5 min at 30 °C and stopped by spotting on P81 phosphocellulose paper. The P81 paper was washed four times in 2 l of 0.452% *o*-phosphoric acid, once in 95% EtOH, and dried in an 80 °C oven. Phosphate incorporation was determined by liquid scintillation counting.

### Western blotting for purified PKGI proteins

Purified PKGI samples were diluted ∼1:100 in KPEB buffer and mixed with 2:1 with 3× SDS-loading buffer containing 300 mM maleimide. Samples were loaded onto 9% SDS-PAGE gels without heating. Separated proteins were transferred to Immobilon, blocked with 5% milk in TBS. Blots were probed with HRP-conjugated anti-Flag antibody at a 1:5000 dilution in 5% milk.

### Analysis of VASP phosphorylation in H9c2(2-1) and C2C12 cells

H9c2(2-1) and C2C12 cells were split into 12-well cluster dishes and 24-h later, wells were treated with 8-CPT-cGMP or H_2_O_2_ as indicated in the figure legends. Cells were lysed in ice cold Buffer A containing 100 mM maleimide. Lysates were cleared by centrifugation and aliquots were added to 3× SDS sample buffer with or without β-mercaptoethanol. Reduced samples were boiled at 100 °C for 5 min before loading on 9% SDS-PAGE gels. Nonreduced samples were loaded onto the gels without boiling. Western blots were performed as described above, using the indicated antibodies.

## Data availability

All supporting data is in the article.

## Supporting information

This article contains supporting information.

## Conflict of interest

The authors declare that they have no conflicts of interest with the contents of this article.
