# Supplementary material for: PKGIα is activated by metal-dependent oxidation in vitro but not in intact cells
Source: J Biol Chem. 2022 Jun 22;298(8):102175. doi: 10.1016/j.jbc.2022.102175 (PMC9293632; doi:10.1016/j.jbc.2022.102175)

Supporting Information

PKGIα is activated by metal-dependent oxidation *in vitro* but not in intact cells

Sahar Aminzai, Tingfei Hu, Renate B. Pilz and Darren E. Casteel

This file contains supplemental figures which show PKGIα dose response data for non-canonical cyclic nucleotides, C2C12 cell data, and demonstrate the reproducibility of the results shown in the main text.

Supplemental Figure Legends

Fig. S1: PKGIα basal activity increases after over-night storage in Flag elution buffer. (A) Newly purified PKGIα was incubated for 1 hour on ice in KPE buffer with the indicated amount of DTT and the level of Cys^{43} crosslinked PKGIα was determined by Western blotting (M = monomeric, D = crosslinked dimeric). (B) Kinase activity in the absence and presence of 10 μM cGMP was measured shortly after purification using an *in vitro* assay. (C, D) The purified PKGIα was stored overnight in elution buffer, and then incubated for one hour with the indicated amounts of DTT in KPE buffer. The amount of crosslinked PKGIα with Cys^{43} oxidized was determined by Western Blotting (C) and kinase activity was measured (D). (E, F) *In vitro* kinase activity of newly purified PKGIα (E) and after overnight storage with different levels of dilution (F). The figure shows data from a single protein preparation with assays performed in triplicate.

Fig. S2: PKGIα activation is prevented in the presence of reducing agents and metal chelators. (A) *In vitro* kinase activity of PKGIα within 1 hour of purification in the absence and presence of 10 μM cGMP. (B) PKGIα activity after over-night incubation in elution buffer alone or in elution buffer with the addition of DTT or EDTA, as indicated. (C) PKGIα activity when freshly prepared and after overnight storage ± 200 pM $CuCl_2$. The figure shows data from a single protein preparation with assays performed in triplicate.

Fig. S3: PKGIα activation is independent of C43S oxidation. (A) The activities of freshly purified wild-type and C43S PKGIα were analyzed by an *in vitro* assay in the absence and presence of 10 μM cGMP. (B) *In vitro* kinase assays performed using the protein preparations from A after overnight storage. The figure shows data from a single protein preparation with assays performed in triplicate.

Fig S4: PKGIα oxidation does not change its K_a for non-canonical cyclic nucleotides. Purified PKGIα was diluted in cold KPE buffer and incubated in the presence or absence of 15 mM DTT for one hour on ice and then used in *in vitro* kinase reactions with increasing amounts of (A) cAMP, (B) cCMP, or (C) cIMP. K_a values were calculated using GraphPad Prism. Western blots show the relative amounts of reduced (-M) and oxidized (-D) PKGIα in the samples used for the reactions.

Fig. S5: Mutation of either Cys^{118} or Cys^{196} reduces oxidation mediated PKGIα activation. (A) Kinase assays performed on wild-type, C118A, and C196V PKGIα immediately after purification. (B) Kinase assays performed after overnight storage. The figure shows data from a single protein preparation with assays performed in triplicate.

Fig. S6: PKGIβ is not highly activated during overnight storage. (A) Kinase assays performed with purified PKGIα and PKGIβ immediately after purification. (B) Kinase assays performed after overnight storage in elution buffer. (C) Kinase assays performed with wild-type and R82F/K83P (RK/FP) PKGIα within 1 hour of purification. (D) Kinase assays performed on the protein preparations shown in C after 20-hour storage in elution buffer at 4°C. The figure shows data from a single protein preparation with assays performed in triplicate.

Fig. S7: Residues throughout the PKGIα autoinhibitory region mediates oxidant-induced activation of PKGIα. (A) Kinase assays performed on wild-type and leucine zipper swapped C1 chimeric PKGI within 1 hour of purification (α/βPKGI = chimeric protein with PKGIα leucine zipper and PKGIβ autoinhibitory loop, β/αPKGI = chimeric protein with PKGIβ leucine zipper and PKGIα autoinhibitory loop. (B) Kinase assays performed using the protein preparations shown in panel A after 20-hour storage in elution buffer at 4°C. (C) Kinase assays performed on wild-type and C2 chimeric PKGI within 1 hour of purification (α/β2PKGI = chimeric PKGI with PKGIα residues N-terminal to the splice site, β/α2PKGI = chimeric protein with PKGIβ residues N-terminal to the splice site. (D) Kinase assays performed using the protein preparations shown in panel C after 20-hour storage in elution buffer at 4°C.

Fig. S8: Testing the effect of acidic residue mutations at PKGIα Cys[118] and PKGIβ Cys[196] on kinase activity. (A) Kinase assays using wild-type PKGIα, wild-type PKGIβ, C118D PKGIα, and C133D PKGIβ. Assays were performed within 1 hour of purification. (B) Kinase assays performed on the protein preparations shown in A after 20-hour storage in elution buffer at 4°C. The figure shows data from a single protein preparation with assays performed in triplicate.

Fig S9: H_2O_2 does not activate PKGIα in C2C12 cells. C2C12 cells were treated with H_2O_2 or 8-CPT-cGMP for 1 hour, as indicated. (A) The amount of Ser[239] phosphorylated VASP (upper panel) and crosslinked/dimeric (oxidized) and momomeric (reduced) PKGI (lower panel) was analyzed by immunoblotting (-D = crosslinked/dimeric and -M = monomeric PKGIα). Equal loading is demonstrated by a non-specific (n.s) band running beneath monomeric PKGIα. (B) Quantification of VASP phospho-Ser[239] from three independent experiments as described in A.

Supplemental Figure 1

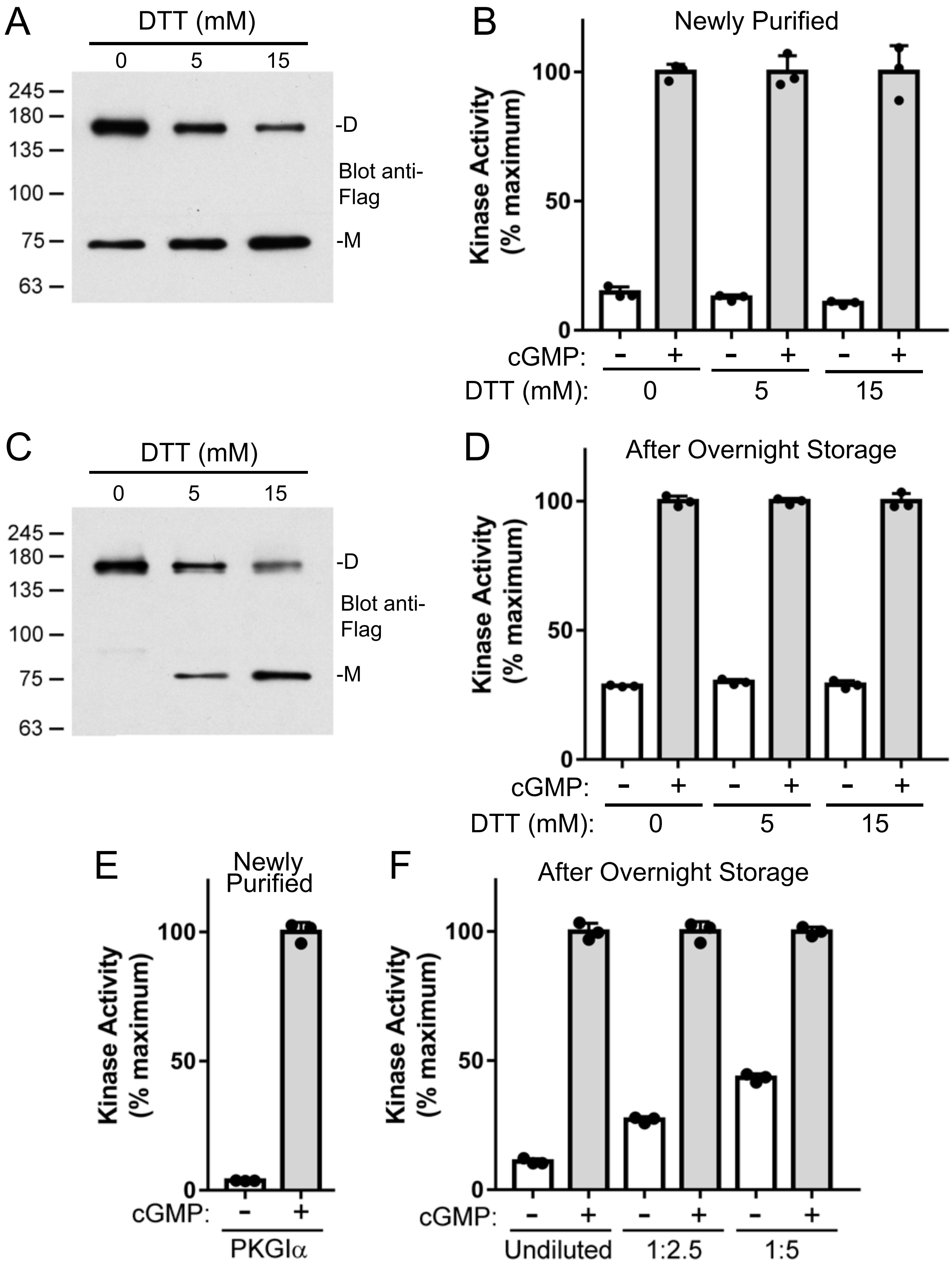

Supplemental Figure 2

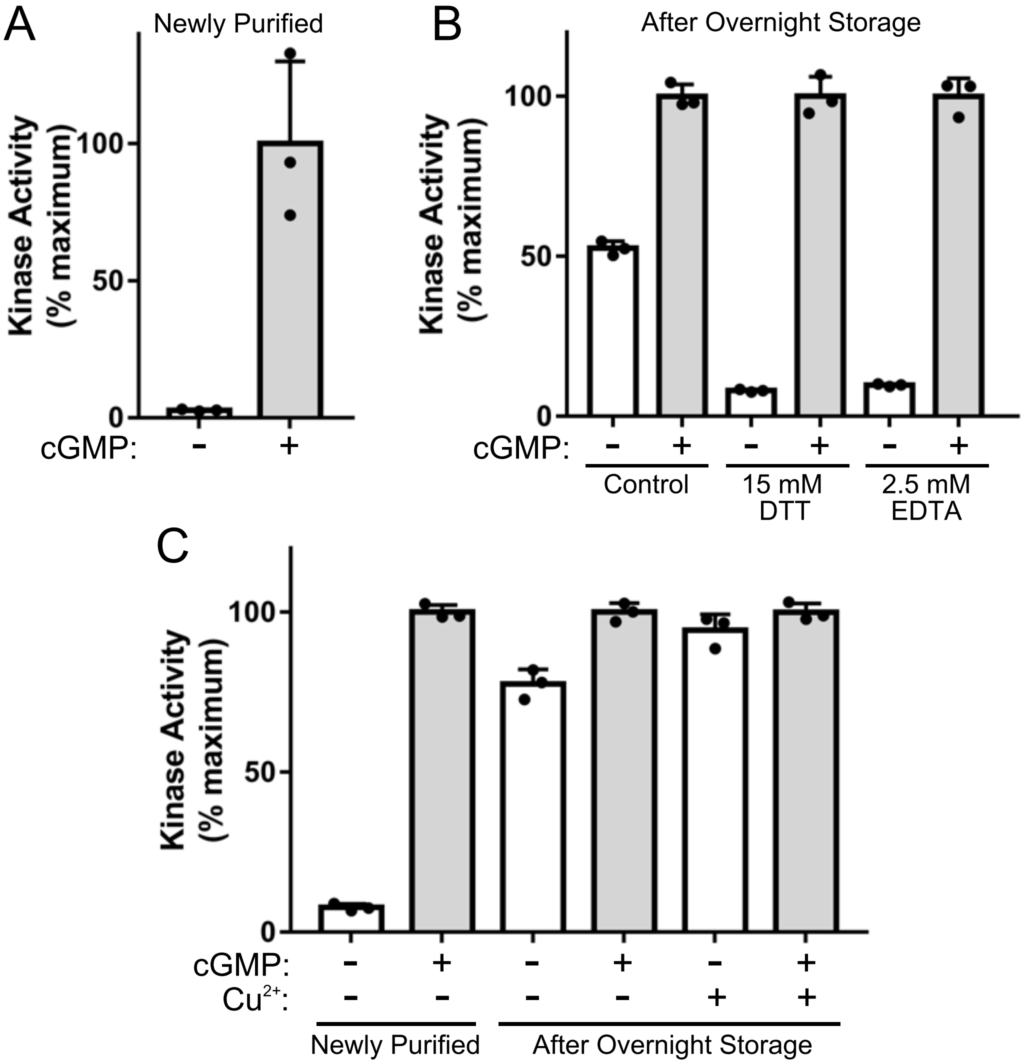

Supplemental Figure 3

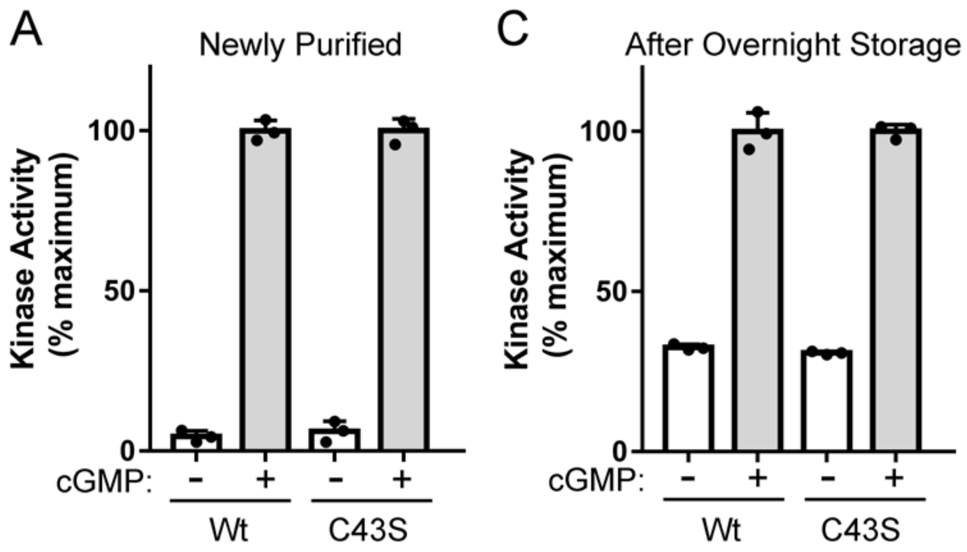

Supplemental Figure 4

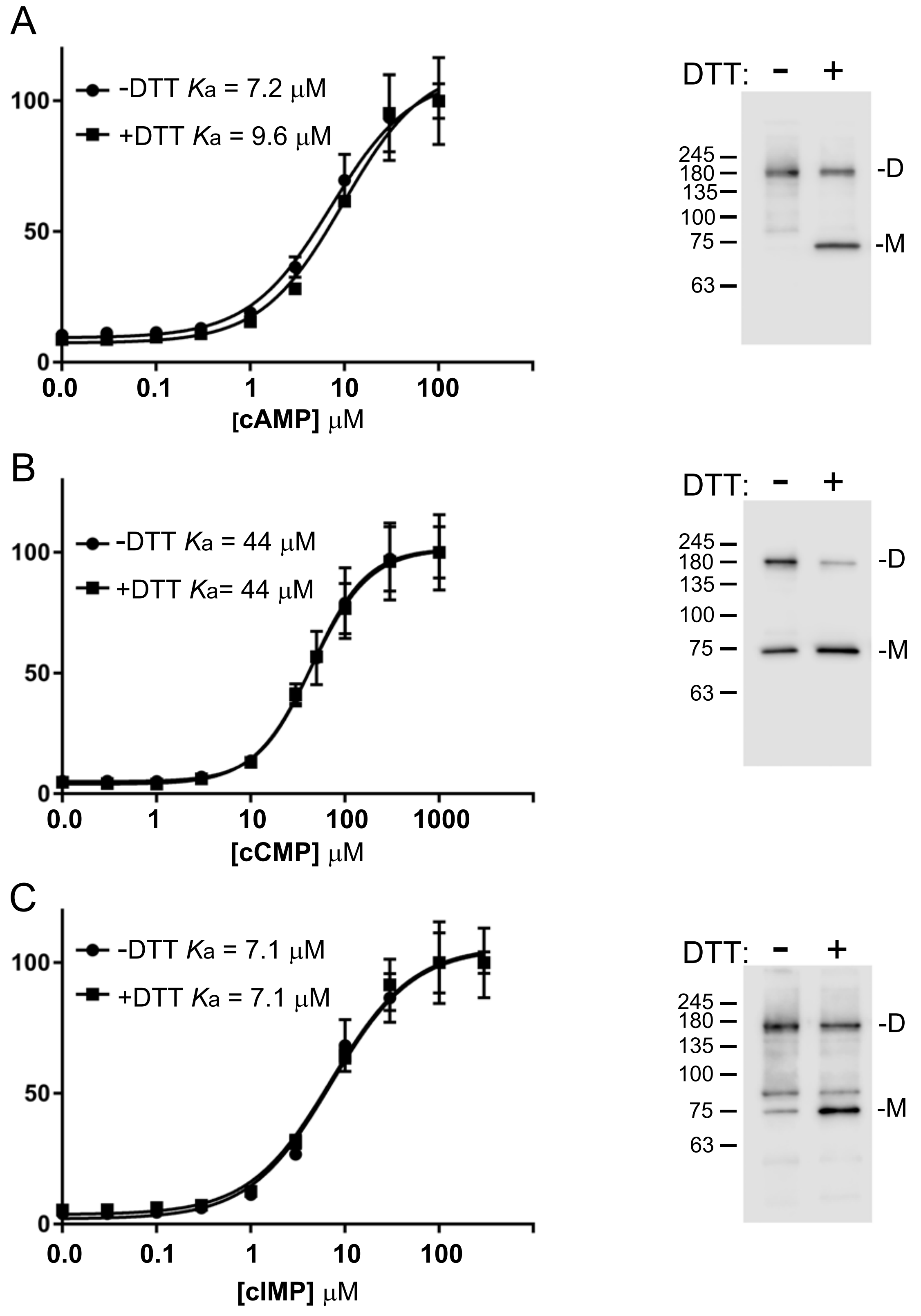

Supplemental Figure 5

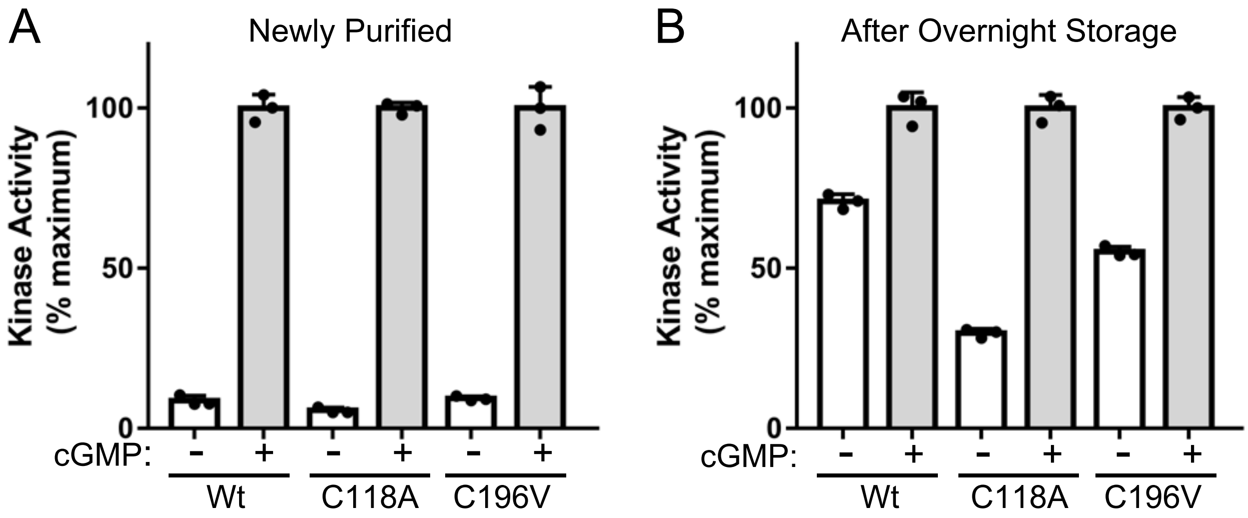

Supplemental Figure 6

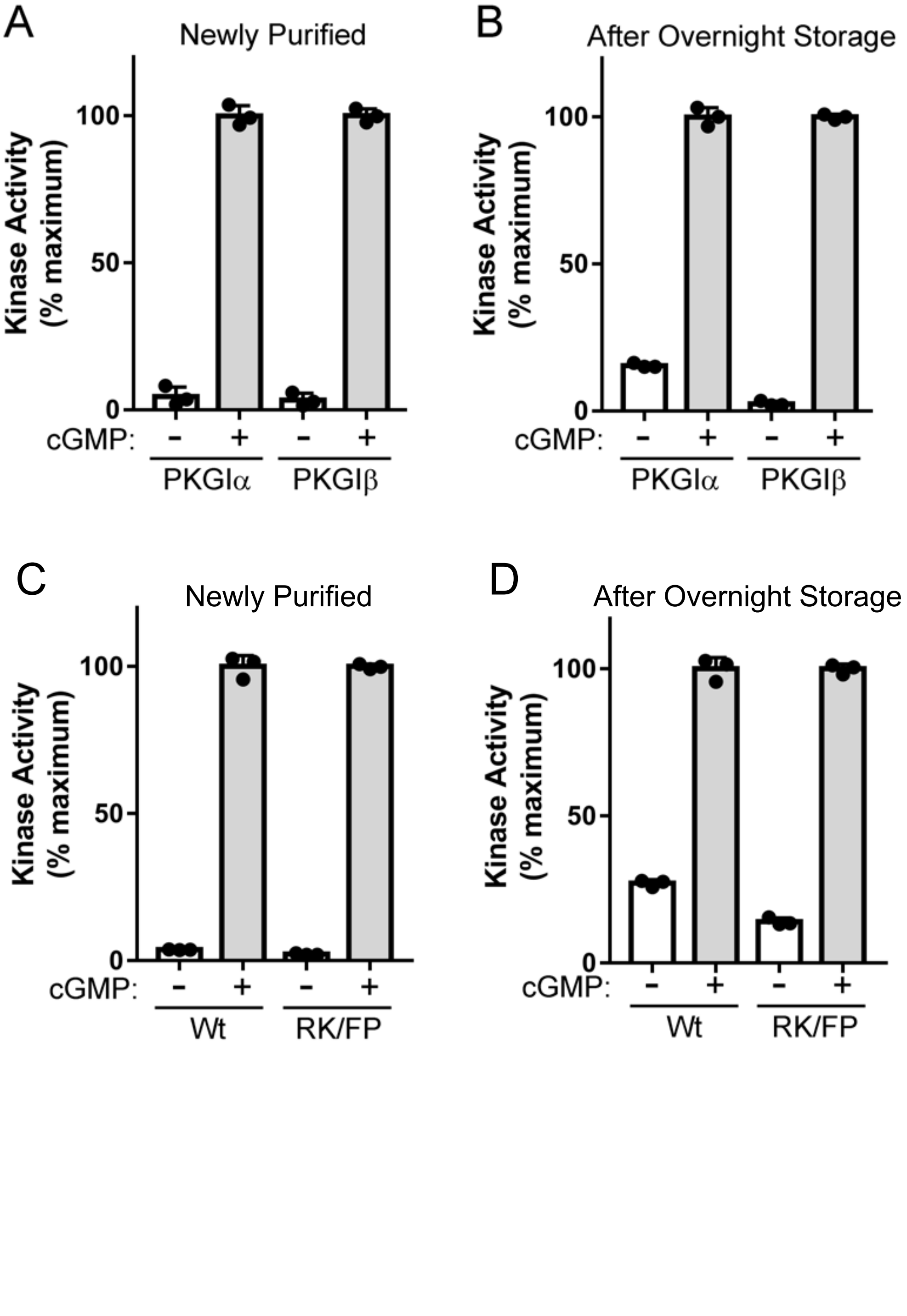

Supplemental Figure 7

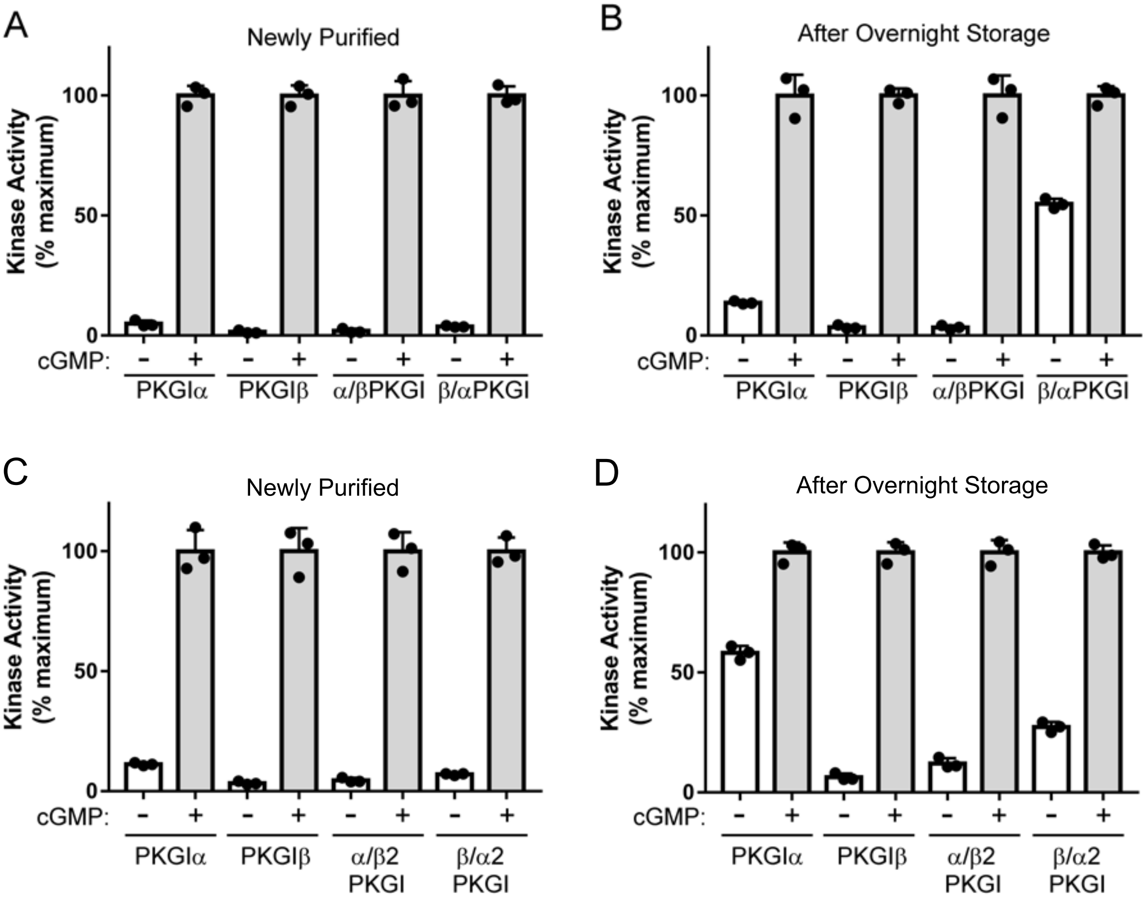

Supplemental Figure 8

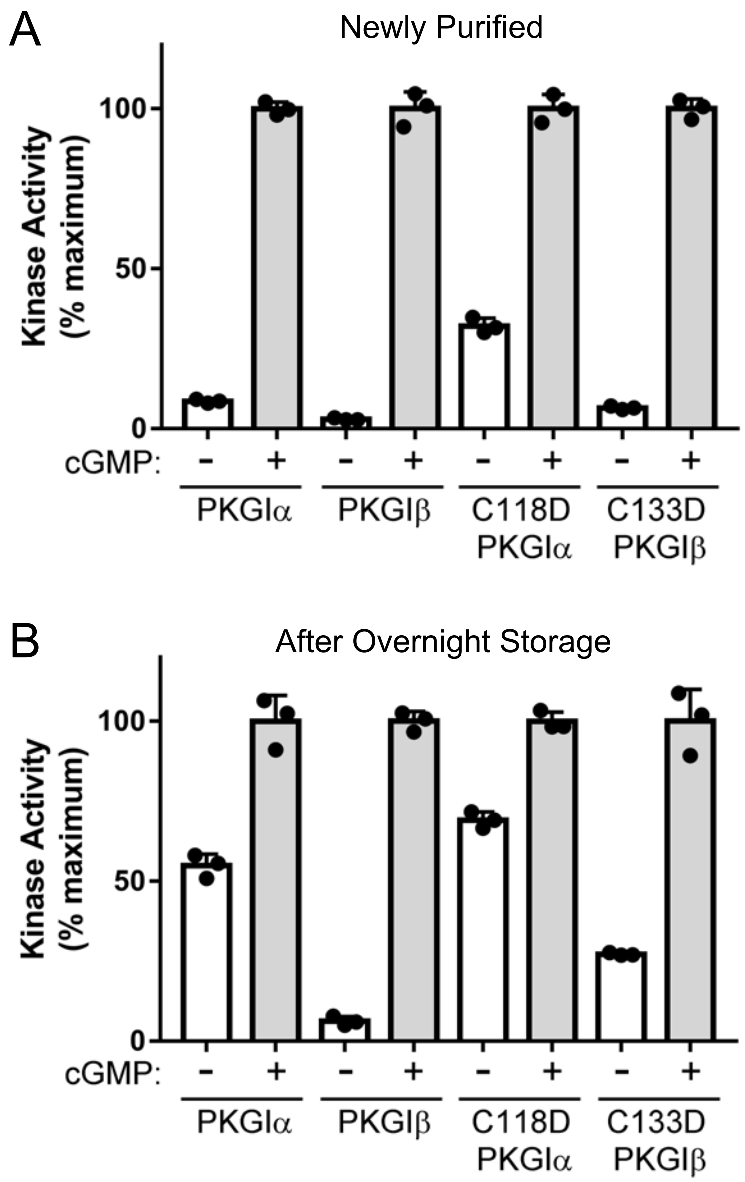

Supplemental Figure 9

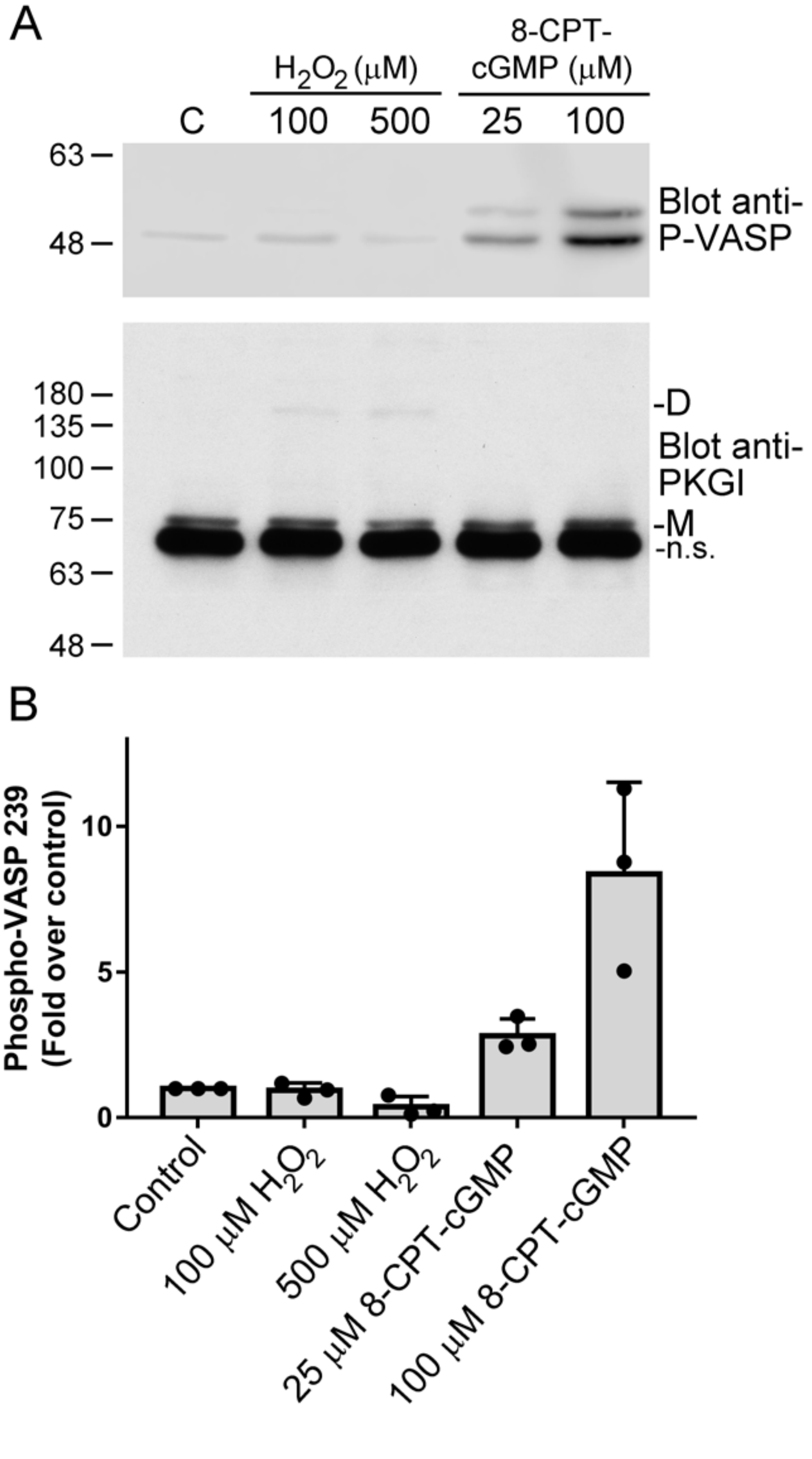

Supplement: Supplemental Figures S1–S9 [file mmc1.pdf]
